# 3-(2-Amino-5-nitro­anilino)-5,5-dimethyl­cyclo­hex-2-en-1-one 0.25-hydrate

**DOI:** 10.1107/S1600536810033933

**Published:** 2010-08-28

**Authors:** Sayed Hasan Mehdi, Rokiah Hashim, Raza Murad Ghalib, Jia Hao Goh, Hoong-Kun Fun

**Affiliations:** a School of Industrial Technology, Universiti Sains Malaysia, 11800 USM, Penang, Malaysia; b X-ray Crystallography Unit, School of Physics, Universiti Sains Malaysia, 11800 USM, Penang, Malaysia

## Abstract

The asymmetric unit of the title compound, C_14_H_17_N_3_O_3_·0.25H_2_O, comprises two independent organic mol­ecules and a water mol­ecule lying on a crystallographic twofold rotation axis with 50% site occupancy. In both independent mol­ecules, the cyclo­hexene rings adopt envelope conformations but superposition of the two molecules shows that the flap atoms point in opposite directions. In the crystal, N—H⋯O and C—H⋯O hydrogen bonds inter­connect adjacent mol­ecules into a three-dimensional network. Weak inter­molecular π–π aromatic stacking inter­actions [centroid–centroid distances = 3.4985 (9) and 3.6630 (9) Å] are also observed.

## Related literature

For general background to (2-amino­phen­yl)amino­cyclo­hexene derivatives, see: Cortés *et al.* (2004 ▶); Tonkikh *et al.* (2004 ▶). For ring conformations and puckering analysis, see: Cremer & Pople (1975 ▶). For related structures, see: Ghalib *et al.* (2010 ▶); Mehdi *et al.* (2010 ▶). For the stability of the temperature controller used in the data collection, see: Cosier & Glazer (1986 ▶).
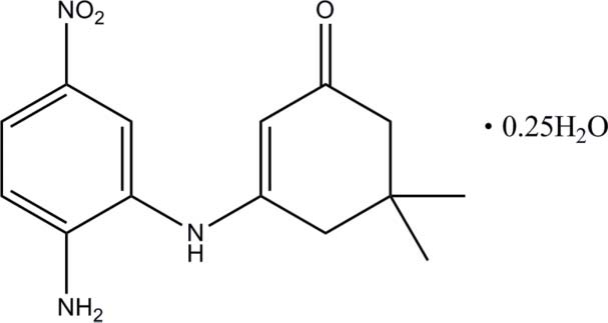


## Experimental

### 

#### Crystal data

C_14_H_17_N_3_O_3_·0.25H_2_O
*M* *_r_* = 279.81
Monoclinic, 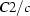

*a* = 18.9043 (1) Å
*b* = 16.7048 (1) Å
*c* = 17.8806 (2) Å
β = 102.443 (1)°
*V* = 5513.93 (8) Å^3^
*Z* = 16
Mo *K*α radiation
μ = 0.10 mm^−1^
*T* = 100 K
0.36 × 0.10 × 0.10 mm

#### Data collection

Bruker SMART APEXII CCD area-detector diffractometer
Absorption correction: multi-scan (*SADABS*; Bruker, 2009 ▶) *T*_min_ = 0.966, *T*_max_ = 0.991
58072 measured reflections
8086 independent reflections
5448 reflections with *I* > 2σ(*I*)
*R*_int_ = 0.072

#### Refinement

*R*[*F* ^2^ > 2σ(*F* ^2^)] = 0.053
*wR*(*F* ^2^) = 0.135
*S* = 1.06
8086 reflections
398 parameters
H atoms treated by a mixture of independent and constrained refinement
Δρ_max_ = 0.25 e Å^−3^
Δρ_min_ = −0.28 e Å^−3^

### 

Data collection: *APEX2* (Bruker, 2009 ▶); cell refinement: *SAINT* (Bruker, 2009 ▶); data reduction: *SAINT*; program(s) used to solve structure: *SHELXTL* (Sheldrick, 2008 ▶); program(s) used to refine structure: *SHELXTL*; molecular graphics: *SHELXTL*; software used to prepare material for publication: *SHELXTL* and *PLATON* (Spek, 2009 ▶).

## Supplementary Material

Crystal structure: contains datablocks global, I. DOI: 10.1107/S1600536810033933/ci5160sup1.cif

Structure factors: contains datablocks I. DOI: 10.1107/S1600536810033933/ci5160Isup2.hkl

Additional supplementary materials:  crystallographic information; 3D view; checkCIF report

## Figures and Tables

**Table 1:** Hydrogen-bond geometry (Å, °)

*D*—H⋯*A*	*D*—H	H⋯*A*	*D*⋯*A*	*D*—H⋯*A*
N1*A*—H1*NA*⋯O1*B*^i^	0.89 (2)	1.99 (2)	2.8678 (17)	172 (2)
N3*A*—H2*NA*⋯O1*A*^ii^	0.90 (2)	2.09 (2)	2.9874 (19)	176 (2)
N3*A*—H3*NA*⋯O2*B*^iii^	0.91 (2)	2.40 (2)	3.258 (2)	156 (2)
N1*B*—H1*NB*⋯O1*W*^iv^	0.89 (2)	2.45 (2)	3.2651 (15)	152 (2)
N3*B*—H2*NB*⋯O1*B*^v^	0.88 (2)	2.05 (2)	2.9161 (19)	169 (2)
N3*B*—H3*NB*⋯O1*A*^vi^	0.88 (2)	2.06 (2)	2.8963 (18)	159 (2)
C8*A*—H8*AA*⋯O2*B*^iii^	0.97	2.55	3.280 (2)	132
C8*B*—H8*BA*⋯O1*W*^iv^	0.97	2.32	3.247 (2)	160

Symmetry codes: (i) 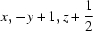; (ii) 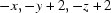; (iii) 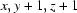; (iv) 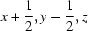; (v) 
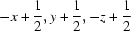
; (vi) 
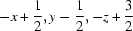
.

**Fig. 1.**
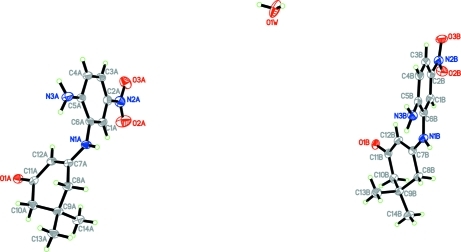
The asymmetric unit of the title compound, showing 50% probability displacement ellipsoids for non-H atoms and the atom-numbering scheme.

**Fig. 2.**
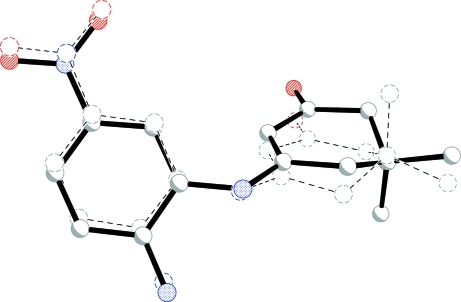
Fit of molecule A (dashed lines) on molecule B (solid lines). H atoms have been omitted for clarity.

**Fig. 3.**
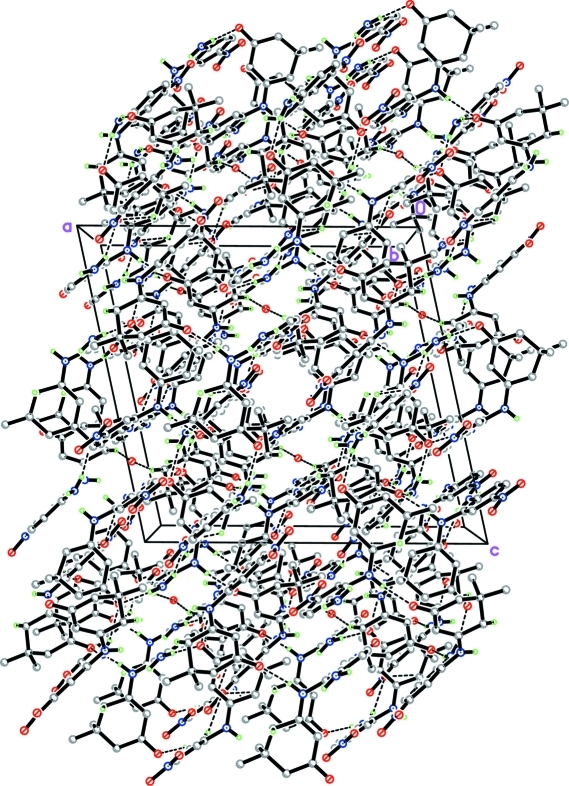
The crystal structure of the title compound, viewed down the b axis. H atoms not involved in hydrogen bonds (dashed lines) have been omitted for clarity.

## supplementary crystallographic information

### Comment

In the recent past many (2-aminophenyl)aminocyclohexene derivatives have been prepared by different methods (Tonkikh *et al.*, 2004; Cortés *et al.*, 2004). Recently we have reported the synthesis of 1,3,3-trimethyl-1,2,3,4-tetrahydropyrido[1,2-*a*]benzimidazol-1-ol by the reaction of dimedone with orthophenylenediamine in acetic acid and ethanol (Mehdi *et al.*, 2010). In this paper we report the synthesis and crystal structure of the title compound by the reaction of dimedone with 4-nitro *o*-phenylenediamine in the presence of acetic acid and ethanol.

The asymmetric unit of the title compound comprises of two 3-[(2-amino-5-nitrophenyl)amino]-5,5-dimethylcyclohex-2-enone molecules (*A* and *B*) and half of a water molecule (Fig. 1). The water molecule lies on a crystallographic twofold rotation axis and the other half of the molecule is generated by the symmetry operation (-x, y, 1/2-z). In both molecules, the cyclohexene rings (C7A-C12A and C7B-C12B) adopt envelope conformations. The puckering parameters are Q = 0.443 (3) Å, θ = 126.3 (2)° and φ = 295.3 (3)° for molecule *A* and Q = 0.448 (2) Å, θ = 51.6 (2)° and φ = 122.2 (3)° for molecule *B*. Atoms C9A and C9B are the flap atoms of C7A-C12A and C7B-C12B cyclohexene rings, respectively, deviating from the mean planes formed through the remaining five atoms by -0.6196 (17) and 0.6286 (15) Å, respectively. The superposition of the non-H atoms of molecules *A* and *B* (Fig. 2) using *XP* in *SHELXTL* (Sheldrick, 2008) shows that the geometries of the two cyclohexene rings are different, with flap atoms in opposite directions. The bond lengths and angles are comparable to those observed in related structures (Ghalib *et al.*, 2010; Mehdi *et al.*, 2010).

In the crystal structure, adjacent molecules are interconnected into a three-dimensional network through N1A—H1NA···O1B, N3A—H2NA···O1A, N3A—H3NA···O2B, N1B—H1NB···O1W, N3B—H2NB···O1B, N3B—H3NB···O1A, C8A—H8AA···O2B, C8B—H8BA···O1W hydrogen bonds (Table 1). The crystal structure is further stabilized by intermolecular aromatic stacking interactions with *Cg*1···*Cg*1^*^ = 3.6630 (19) Å and *Cg*2···*Cg*2^$^ 3.4985 (9) Å [symmetry codes: (*) -x, y, 3/2-z; ($) 1/2-x, 1/2-y, -z] where *Cg*1 and *Cg*2 are centroids of the C1A-C6A and C1B-C6B benzene rings, respectively.

### Experimental

A mixture of 4-nitro *o*-phenylenediamine (0.153 g) and dimedone (0.140 g) in a 1:1 molar ratio was refluxed in a mixture of acetic acid and ethanol (1:1 v/v) for 3 h. The solid settled in the reaction mixture was filtered and crystallized in ethanol to furnish orange-coloured single crystals of the title compound (100 mg, m.p. 481 K). The melting point was taken using the Thermo Fisher digital melting point apparatus of IA9000 series.

### Refinement

H atoms bound to O and N atoms were located in a difference Fourier map and refined freely [O–H = 0.83 (2) Å, range of N–H = 0.88 (2)–0.91 (2) Å]. The remaining H atoms were placed in their calculated positions, with C–H = 0.93–0.97 Å, and refined using a riding model, with *U*_iso_ = 1.2 or 1.5 *U*_eq_(C). A rotating group model was applied to the methyl groups.

### Crystal data

**Table:**

C_14_H_17_N_3_O_3_·0.25H_2_O	*F*(000) = 2376
*M**_r_* = 279.81	*D*_x_ = 1.348 Mg m^−^^3^
Monoclinic, *C*2/*c*	Mo *K*α radiation, λ = 0.71073 Å
Hall symbol: -C 2yc	Cell parameters from 6071 reflections
*a* = 18.9043 (1) Å	θ = 2.2–24.2°
*b* = 16.7048 (1) Å	µ = 0.10 mm^−^^1^
*c* = 17.8806 (2) Å	*T* = 100 K
β = 102.443 (1)°	Needle, orange
*V* = 5513.93 (8) Å^3^	0.36 × 0.10 × 0.10 mm
*Z* = 16	

### Data collection

**Table:**

Bruker SMART APEXII CCD area-detector diffractometer	8086 independent reflections
Radiation source: fine-focus sealed tube	5448 reflections with *I* > 2σ(*I*)
graphite	*R*_int_ = 0.072
φ and ω scans	θ_max_ = 30.1°, θ_min_ = 1.6°
Absorption correction: multi-scan (*SADABS*; Bruker, 2009)	*h* = −26→26
*T*_min_ = 0.966, *T*_max_ = 0.991	*k* = −23→23
58072 measured reflections	*l* = −25→25

### Refinement

**Table:**

Refinement on *F*^2^	Primary atom site location: structure-invariant direct methods
Least-squares matrix: full	Secondary atom site location: difference Fourier map
*R*[*F*^2^ > 2σ(*F*^2^)] = 0.053	Hydrogen site location: inferred from neighbouring sites
*wR*(*F*^2^) = 0.135	H atoms treated by a mixture of independent and constrained refinement
*S* = 1.06	*w* = 1/[σ^2^(*F*_o_^2^) + (0.0547*P*)^2^ + 2.5234*P*] where *P* = (*F*_o_^2^ + 2*F*_c_^2^)/3
8086 reflections	(Δ/σ)_max_ = 0.001
398 parameters	Δρ_max_ = 0.25 e Å^−^^3^
0 restraints	Δρ_min_ = −0.28 e Å^−^^3^

### Special details

**Table:**

Experimental. The crystal was placed in the cold stream of an Oxford Cryosystems Cobra open-flow nitrogen cryostat (Cosier & Glazer, 1986) operating at 100.0 (1)K.
Geometry. All esds (except the esd in the dihedral angle between two l.s. planes) are estimated using the full covariance matrix. The cell esds are taken into account individually in the estimation of esds in distances, angles and torsion angles; correlations between esds in cell parameters are only used when they are defined by crystal symmetry. An approximate (isotropic) treatment of cell esds is used for estimating esds involving l.s. planes.
Refinement. Refinement of F^2^ against ALL reflections. The weighted R-factor wR and goodness of fit S are based on F^2^, conventional R-factors R are based on F, with F set to zero for negative F^2^. The threshold expression of F^2^ > 2sigma(F^2^) is used only for calculating R-factors(gt) etc. and is not relevant to the choice of reflections for refinement. R-factors based on F^2^ are statistically about twice as large as those based on F, and R- factors based on ALL data will be even larger.

### Fractional atomic coordinates and isotropic or equivalent isotropic displacement parameters (Å^2^)

**Table:**

	*x*	*y*	*z*	*U*_iso_*/*U*_eq_	
O1A	0.10407 (6)	0.88026 (7)	1.18606 (6)	0.0250 (2)	
O2A	−0.00894 (8)	0.63989 (8)	0.87992 (10)	0.0560 (4)	
O3A	−0.10784 (7)	0.66814 (8)	0.79713 (7)	0.0428 (3)	
N1A	0.12683 (7)	0.89926 (8)	0.92807 (7)	0.0233 (3)	
N2A	−0.04981 (8)	0.68814 (9)	0.83987 (9)	0.0345 (3)	
N3A	0.02428 (8)	1.01243 (9)	0.86062 (9)	0.0296 (3)	
C1A	0.03700 (8)	0.79493 (10)	0.88679 (9)	0.0247 (3)	
H1AA	0.0693	0.7567	0.9120	0.030*	
C2A	−0.03046 (9)	0.77211 (10)	0.84420 (9)	0.0261 (3)	
C3A	−0.07831 (8)	0.82840 (10)	0.80490 (9)	0.0263 (3)	
H3AA	−0.1221	0.8121	0.7742	0.032*	
C4A	−0.06102 (8)	0.90791 (10)	0.81135 (9)	0.0260 (3)	
H4AA	−0.0940	0.9455	0.7862	0.031*	
C5A	0.00592 (8)	0.93374 (9)	0.85545 (8)	0.0226 (3)	
C6A	0.05544 (8)	0.87464 (9)	0.89116 (8)	0.0213 (3)	
C7A	0.15228 (8)	0.90426 (9)	1.00454 (8)	0.0211 (3)	
C8A	0.22892 (8)	0.93365 (10)	1.02831 (9)	0.0240 (3)	
H8AA	0.2287	0.9917	1.0291	0.029*	
H8AB	0.2557	0.9168	0.9905	0.029*	
C9A	0.26804 (8)	0.90297 (10)	1.10715 (9)	0.0237 (3)	
C10A	0.21925 (8)	0.91886 (10)	1.16357 (9)	0.0264 (3)	
H10A	0.2395	0.8914	1.2112	0.032*	
H10B	0.2201	0.9758	1.1745	0.032*	
C11A	0.14128 (8)	0.89301 (9)	1.13669 (9)	0.0219 (3)	
C12A	0.11152 (8)	0.88643 (9)	1.05722 (8)	0.0219 (3)	
H12A	0.0638	0.8698	1.0405	0.026*	
C13A	0.33996 (9)	0.94785 (11)	1.13176 (10)	0.0305 (4)	
H13A	0.3308	1.0043	1.1327	0.046*	
H13B	0.3701	0.9370	1.0960	0.046*	
H13C	0.3641	0.9304	1.1819	0.046*	
C14A	0.28313 (9)	0.81308 (10)	1.10315 (10)	0.0324 (4)	
H14A	0.3104	0.7951	1.1519	0.049*	
H14B	0.3104	0.8034	1.0645	0.049*	
H14C	0.2381	0.7845	1.0905	0.049*	
O1B	0.23010 (6)	0.05262 (7)	0.34053 (6)	0.0273 (3)	
O2B	0.12655 (7)	0.09396 (7)	0.01137 (7)	0.0373 (3)	
O3B	0.06840 (7)	0.19146 (8)	−0.05509 (7)	0.0393 (3)	
N1B	0.34019 (7)	0.22183 (8)	0.18568 (8)	0.0239 (3)	
N2B	0.11744 (8)	0.16604 (9)	−0.00325 (8)	0.0299 (3)	
N3B	0.31623 (8)	0.38637 (9)	0.15638 (8)	0.0262 (3)	
C1B	0.22746 (8)	0.19536 (10)	0.09317 (9)	0.0247 (3)	
H1BA	0.2346	0.1407	0.1010	0.030*	
C2B	0.16734 (8)	0.22303 (10)	0.04083 (9)	0.0250 (3)	
C3B	0.15569 (9)	0.30448 (10)	0.02732 (9)	0.0276 (3)	
H3BA	0.1150	0.3224	−0.0076	0.033*	
C4B	0.20513 (9)	0.35776 (10)	0.06638 (9)	0.0264 (3)	
H4BA	0.1980	0.4122	0.0569	0.032*	
C5B	0.26681 (8)	0.33224 (9)	0.12075 (8)	0.0234 (3)	
C6B	0.27680 (8)	0.24929 (9)	0.13365 (9)	0.0231 (3)	
C7B	0.34175 (8)	0.16652 (9)	0.24196 (9)	0.0220 (3)	
C8B	0.41600 (8)	0.13787 (9)	0.28068 (9)	0.0243 (3)	
H8BA	0.4500	0.1816	0.2823	0.029*	
H8BB	0.4304	0.0954	0.2501	0.029*	
C9B	0.42129 (8)	0.10673 (9)	0.36246 (9)	0.0243 (3)	
C10B	0.35921 (9)	0.04699 (9)	0.36023 (9)	0.0259 (3)	
H10C	0.3693	−0.0011	0.3339	0.031*	
H10D	0.3578	0.0321	0.4123	0.031*	
C11B	0.28568 (8)	0.07847 (9)	0.32102 (9)	0.0237 (3)	
C12B	0.28102 (8)	0.13614 (10)	0.26177 (9)	0.0252 (3)	
H12B	0.2357	0.1537	0.2358	0.030*	
C13B	0.41499 (10)	0.17608 (11)	0.41657 (10)	0.0330 (4)	
H13D	0.4195	0.1559	0.4677	0.049*	
H13E	0.3687	0.2016	0.4002	0.049*	
H13F	0.4528	0.2142	0.4158	0.049*	
C14B	0.49440 (9)	0.06534 (11)	0.38949 (10)	0.0324 (4)	
H14D	0.4997	0.0491	0.4419	0.049*	
H14E	0.5326	0.1018	0.3853	0.049*	
H14F	0.4968	0.0191	0.3582	0.049*	
O1W	0.0000	0.80007 (11)	0.2500	0.0446 (5)	
H1W1	0.0200 (13)	0.8311 (14)	0.2244 (13)	0.055 (7)*	
H1NA	0.1562 (11)	0.9117 (12)	0.8972 (11)	0.037 (5)*	
H2NA	−0.0127 (12)	1.0468 (13)	0.8460 (12)	0.047 (6)*	
H3NA	0.0637 (11)	1.0285 (12)	0.8965 (11)	0.037 (5)*	
H1NB	0.3824 (12)	0.2453 (13)	0.1855 (12)	0.047 (6)*	
H2NB	0.3020 (11)	0.4365 (13)	0.1504 (11)	0.035 (5)*	
H3NB	0.3455 (10)	0.3735 (11)	0.1998 (11)	0.030 (5)*	

### Atomic displacement parameters (Å^2^)

**Table:**

	*U*^11^	*U*^22^	*U*^33^	*U*^12^	*U*^13^	*U*^23^
O1A	0.0203 (5)	0.0317 (6)	0.0243 (5)	−0.0009 (4)	0.0073 (4)	0.0030 (4)
O2A	0.0471 (9)	0.0294 (8)	0.0832 (11)	0.0007 (7)	−0.0043 (8)	0.0026 (7)
O3A	0.0422 (8)	0.0446 (8)	0.0392 (7)	−0.0179 (6)	0.0038 (6)	−0.0100 (6)
N1A	0.0159 (6)	0.0323 (7)	0.0212 (6)	−0.0010 (5)	0.0031 (5)	0.0040 (5)
N2A	0.0322 (8)	0.0323 (8)	0.0385 (8)	−0.0055 (6)	0.0067 (7)	−0.0054 (6)
N3A	0.0222 (7)	0.0276 (8)	0.0365 (8)	0.0030 (6)	0.0010 (6)	0.0021 (6)
C1A	0.0213 (8)	0.0273 (8)	0.0256 (8)	0.0027 (6)	0.0056 (6)	0.0010 (6)
C2A	0.0235 (8)	0.0288 (8)	0.0266 (8)	−0.0029 (6)	0.0069 (6)	−0.0049 (6)
C3A	0.0180 (7)	0.0374 (9)	0.0228 (8)	−0.0009 (6)	0.0026 (6)	−0.0036 (6)
C4A	0.0199 (8)	0.0328 (9)	0.0242 (8)	0.0054 (6)	0.0026 (6)	0.0026 (6)
C5A	0.0190 (7)	0.0287 (8)	0.0203 (7)	0.0017 (6)	0.0049 (6)	0.0015 (6)
C6A	0.0162 (7)	0.0292 (8)	0.0183 (7)	−0.0001 (6)	0.0031 (5)	−0.0004 (6)
C7A	0.0167 (7)	0.0223 (7)	0.0234 (7)	0.0013 (6)	0.0022 (6)	0.0017 (6)
C8A	0.0169 (7)	0.0301 (8)	0.0252 (8)	−0.0026 (6)	0.0050 (6)	0.0022 (6)
C9A	0.0168 (7)	0.0306 (8)	0.0229 (7)	−0.0009 (6)	0.0025 (6)	0.0020 (6)
C10A	0.0196 (8)	0.0359 (9)	0.0234 (8)	−0.0052 (7)	0.0039 (6)	−0.0014 (6)
C11A	0.0183 (7)	0.0221 (8)	0.0256 (8)	0.0019 (6)	0.0054 (6)	0.0019 (6)
C12A	0.0151 (7)	0.0243 (8)	0.0259 (8)	−0.0010 (6)	0.0035 (6)	0.0017 (6)
C13A	0.0175 (8)	0.0447 (10)	0.0277 (8)	−0.0059 (7)	0.0016 (6)	−0.0023 (7)
C14A	0.0227 (8)	0.0339 (10)	0.0394 (10)	0.0035 (7)	0.0038 (7)	0.0039 (7)
O1B	0.0259 (6)	0.0278 (6)	0.0315 (6)	−0.0050 (5)	0.0136 (5)	−0.0037 (5)
O2B	0.0386 (7)	0.0312 (7)	0.0390 (7)	−0.0043 (5)	0.0018 (6)	0.0000 (5)
O3B	0.0266 (7)	0.0449 (8)	0.0406 (7)	0.0076 (6)	−0.0053 (5)	−0.0044 (6)
N1B	0.0192 (7)	0.0240 (7)	0.0290 (7)	−0.0004 (5)	0.0064 (5)	0.0032 (5)
N2B	0.0255 (7)	0.0338 (8)	0.0303 (7)	0.0007 (6)	0.0058 (6)	−0.0026 (6)
N3B	0.0298 (8)	0.0226 (7)	0.0254 (7)	0.0016 (6)	0.0042 (6)	0.0010 (5)
C1B	0.0250 (8)	0.0233 (8)	0.0273 (8)	0.0023 (6)	0.0090 (6)	0.0003 (6)
C2B	0.0223 (8)	0.0306 (8)	0.0228 (8)	0.0006 (6)	0.0066 (6)	−0.0023 (6)
C3B	0.0264 (8)	0.0332 (9)	0.0236 (8)	0.0072 (7)	0.0060 (6)	0.0010 (6)
C4B	0.0307 (9)	0.0249 (8)	0.0246 (8)	0.0059 (7)	0.0081 (7)	0.0015 (6)
C5B	0.0251 (8)	0.0249 (8)	0.0219 (7)	0.0014 (6)	0.0090 (6)	−0.0011 (6)
C6B	0.0211 (7)	0.0259 (8)	0.0233 (7)	0.0026 (6)	0.0068 (6)	0.0008 (6)
C7B	0.0216 (7)	0.0196 (7)	0.0255 (8)	0.0014 (6)	0.0066 (6)	−0.0004 (6)
C8B	0.0188 (7)	0.0232 (8)	0.0319 (8)	−0.0004 (6)	0.0075 (6)	0.0021 (6)
C9B	0.0218 (8)	0.0217 (8)	0.0291 (8)	0.0004 (6)	0.0054 (6)	0.0024 (6)
C10B	0.0263 (8)	0.0224 (8)	0.0301 (8)	−0.0015 (6)	0.0082 (7)	0.0012 (6)
C11B	0.0232 (8)	0.0229 (8)	0.0266 (8)	−0.0034 (6)	0.0090 (6)	−0.0058 (6)
C12B	0.0189 (7)	0.0277 (8)	0.0295 (8)	0.0015 (6)	0.0065 (6)	0.0015 (6)
C13B	0.0299 (9)	0.0326 (9)	0.0357 (9)	−0.0025 (7)	0.0055 (7)	−0.0053 (7)
C14B	0.0252 (9)	0.0332 (9)	0.0382 (10)	0.0021 (7)	0.0054 (7)	0.0070 (7)
O1W	0.0529 (12)	0.0203 (9)	0.0751 (15)	0.000	0.0454 (12)	0.000

### Geometric parameters (Å, °)

**Table:**

O1A—C11A	1.2597 (17)	O2B—N2B	1.2363 (18)
O2A—N2A	1.233 (2)	O3B—N2B	1.2363 (18)
O3A—N2A	1.2400 (19)	N1B—C7B	1.3617 (19)
N1A—C7A	1.3507 (19)	N1B—C6B	1.425 (2)
N1A—C6A	1.4290 (19)	N1B—H1NB	0.89 (2)
N1A—H1NA	0.89 (2)	N2B—C2B	1.448 (2)
N2A—C2A	1.448 (2)	N3B—C5B	1.357 (2)
N3A—C5A	1.358 (2)	N3B—H2NB	0.88 (2)
N3A—H2NA	0.90 (2)	N3B—H3NB	0.88 (2)
N3A—H3NA	0.91 (2)	C1B—C6B	1.384 (2)
C1A—C6A	1.374 (2)	C1B—C2B	1.387 (2)
C1A—C2A	1.390 (2)	C1B—H1BA	0.93
C1A—H1AA	0.93	C2B—C3B	1.391 (2)
C2A—C3A	1.387 (2)	C3B—C4B	1.368 (2)
C3A—C4A	1.367 (2)	C3B—H3BA	0.93
C3A—H3AA	0.93	C4B—C5B	1.413 (2)
C4A—C5A	1.407 (2)	C4B—H4BA	0.93
C4A—H4AA	0.93	C5B—C6B	1.411 (2)
C5A—C6A	1.415 (2)	C7B—C12B	1.370 (2)
C7A—C12A	1.372 (2)	C7B—C8B	1.503 (2)
C7A—C8A	1.502 (2)	C8B—C9B	1.535 (2)
C8A—C9A	1.532 (2)	C8B—H8BA	0.97
C8A—H8AA	0.97	C8B—H8BB	0.97
C8A—H8AB	0.97	C9B—C14B	1.528 (2)
C9A—C10A	1.530 (2)	C9B—C13B	1.530 (2)
C9A—C13A	1.532 (2)	C9B—C10B	1.534 (2)
C9A—C14A	1.533 (2)	C10B—C11B	1.510 (2)
C10A—C11A	1.511 (2)	C10B—H10C	0.97
C10A—H10A	0.97	C10B—H10D	0.97
C10A—H10B	0.97	C11B—C12B	1.420 (2)
C11A—C12A	1.415 (2)	C12B—H12B	0.93
C12A—H12A	0.93	C13B—H13D	0.96
C13A—H13A	0.96	C13B—H13E	0.96
C13A—H13B	0.96	C13B—H13F	0.96
C13A—H13C	0.96	C14B—H14D	0.96
C14A—H14A	0.96	C14B—H14E	0.96
C14A—H14B	0.96	C14B—H14F	0.96
C14A—H14C	0.96	O1W—H1W1	0.83 (2)
O1B—C11B	1.2531 (18)		
			
C7A—N1A—C6A	125.48 (13)	C7B—N1B—C6B	125.59 (13)
C7A—N1A—H1NA	118.7 (13)	C7B—N1B—H1NB	115.2 (14)
C6A—N1A—H1NA	115.8 (13)	C6B—N1B—H1NB	119.0 (14)
O2A—N2A—O3A	122.83 (16)	O2B—N2B—O3B	122.74 (14)
O2A—N2A—C2A	118.86 (15)	O2B—N2B—C2B	118.71 (14)
O3A—N2A—C2A	118.29 (15)	O3B—N2B—C2B	118.52 (14)
C5A—N3A—H2NA	115.3 (14)	C5B—N3B—H2NB	114.6 (13)
C5A—N3A—H3NA	119.4 (12)	C5B—N3B—H3NB	119.5 (12)
H2NA—N3A—H3NA	118.9 (19)	H2NB—N3B—H3NB	117.3 (18)
C6A—C1A—C2A	119.23 (15)	C6B—C1B—C2B	119.88 (15)
C6A—C1A—H1AA	120.4	C6B—C1B—H1BA	120.1
C2A—C1A—H1AA	120.4	C2B—C1B—H1BA	120.1
C3A—C2A—C1A	120.86 (15)	C1B—C2B—C3B	121.28 (15)
C3A—C2A—N2A	120.02 (15)	C1B—C2B—N2B	119.42 (15)
C1A—C2A—N2A	119.11 (15)	C3B—C2B—N2B	119.23 (14)
C4A—C3A—C2A	119.89 (15)	C4B—C3B—C2B	118.85 (15)
C4A—C3A—H3AA	120.1	C4B—C3B—H3BA	120.6
C2A—C3A—H3AA	120.1	C2B—C3B—H3BA	120.6
C3A—C4A—C5A	121.00 (15)	C3B—C4B—C5B	121.77 (15)
C3A—C4A—H4AA	119.5	C3B—C4B—H4BA	119.1
C5A—C4A—H4AA	119.5	C5B—C4B—H4BA	119.1
N3A—C5A—C4A	121.40 (15)	N3B—C5B—C6B	121.57 (15)
N3A—C5A—C6A	120.68 (14)	N3B—C5B—C4B	120.31 (15)
C4A—C5A—C6A	117.83 (14)	C6B—C5B—C4B	118.05 (14)
C1A—C6A—C5A	121.02 (14)	C1B—C6B—C5B	120.16 (14)
C1A—C6A—N1A	120.51 (14)	C1B—C6B—N1B	120.61 (14)
C5A—C6A—N1A	118.36 (14)	C5B—C6B—N1B	119.14 (14)
N1A—C7A—C12A	123.45 (14)	N1B—C7B—C12B	123.83 (14)
N1A—C7A—C8A	114.73 (13)	N1B—C7B—C8B	115.19 (13)
C12A—C7A—C8A	121.79 (13)	C12B—C7B—C8B	120.95 (14)
C7A—C8A—C9A	113.34 (12)	C7B—C8B—C9B	114.16 (12)
C7A—C8A—H8AA	108.9	C7B—C8B—H8BA	108.7
C9A—C8A—H8AA	108.9	C9B—C8B—H8BA	108.7
C7A—C8A—H8AB	108.9	C7B—C8B—H8BB	108.7
C9A—C8A—H8AB	108.9	C9B—C8B—H8BB	108.7
H8AA—C8A—H8AB	107.7	H8BA—C8B—H8BB	107.6
C10A—C9A—C13A	110.46 (13)	C14B—C9B—C13B	109.32 (14)
C10A—C9A—C8A	107.98 (12)	C14B—C9B—C10B	110.40 (13)
C13A—C9A—C8A	108.86 (13)	C13B—C9B—C10B	110.22 (13)
C10A—C9A—C14A	110.21 (13)	C14B—C9B—C8B	108.88 (13)
C13A—C9A—C14A	109.28 (13)	C13B—C9B—C8B	110.36 (13)
C8A—C9A—C14A	110.03 (13)	C10B—C9B—C8B	107.65 (13)
C11A—C10A—C9A	115.21 (13)	C11B—C10B—C9B	114.07 (13)
C11A—C10A—H10A	108.5	C11B—C10B—H10C	108.7
C9A—C10A—H10A	108.5	C9B—C10B—H10C	108.7
C11A—C10A—H10B	108.5	C11B—C10B—H10D	108.7
C9A—C10A—H10B	108.5	C9B—C10B—H10D	108.7
H10A—C10A—H10B	107.5	H10C—C10B—H10D	107.6
O1A—C11A—C12A	121.95 (14)	O1B—C11B—C12B	121.36 (15)
O1A—C11A—C10A	118.62 (13)	O1B—C11B—C10B	119.60 (14)
C12A—C11A—C10A	119.40 (13)	C12B—C11B—C10B	119.04 (13)
C7A—C12A—C11A	120.79 (14)	C7B—C12B—C11B	121.56 (15)
C7A—C12A—H12A	119.6	C7B—C12B—H12B	119.2
C11A—C12A—H12A	119.6	C11B—C12B—H12B	119.2
C9A—C13A—H13A	109.5	C9B—C13B—H13D	109.5
C9A—C13A—H13B	109.5	C9B—C13B—H13E	109.5
H13A—C13A—H13B	109.5	H13D—C13B—H13E	109.5
C9A—C13A—H13C	109.5	C9B—C13B—H13F	109.5
H13A—C13A—H13C	109.5	H13D—C13B—H13F	109.5
H13B—C13A—H13C	109.5	H13E—C13B—H13F	109.5
C9A—C14A—H14A	109.5	C9B—C14B—H14D	109.5
C9A—C14A—H14B	109.5	C9B—C14B—H14E	109.5
H14A—C14A—H14B	109.5	H14D—C14B—H14E	109.5
C9A—C14A—H14C	109.5	C9B—C14B—H14F	109.5
H14A—C14A—H14C	109.5	H14D—C14B—H14F	109.5
H14B—C14A—H14C	109.5	H14E—C14B—H14F	109.5
			
C6A—C1A—C2A—C3A	1.5 (2)	C6B—C1B—C2B—C3B	0.7 (2)
C6A—C1A—C2A—N2A	−179.83 (14)	C6B—C1B—C2B—N2B	177.76 (13)
O2A—N2A—C2A—C3A	−174.43 (16)	O2B—N2B—C2B—C1B	5.8 (2)
O3A—N2A—C2A—C3A	4.0 (2)	O3B—N2B—C2B—C1B	−172.66 (14)
O2A—N2A—C2A—C1A	6.9 (2)	O2B—N2B—C2B—C3B	−177.04 (14)
O3A—N2A—C2A—C1A	−174.71 (14)	O3B—N2B—C2B—C3B	4.5 (2)
C1A—C2A—C3A—C4A	−3.8 (2)	C1B—C2B—C3B—C4B	0.5 (2)
N2A—C2A—C3A—C4A	177.60 (14)	N2B—C2B—C3B—C4B	−176.64 (14)
C2A—C3A—C4A—C5A	2.1 (2)	C2B—C3B—C4B—C5B	−1.1 (2)
C3A—C4A—C5A—N3A	178.34 (15)	C3B—C4B—C5B—N3B	177.49 (14)
C3A—C4A—C5A—C6A	1.7 (2)	C3B—C4B—C5B—C6B	0.5 (2)
C2A—C1A—C6A—C5A	2.4 (2)	C2B—C1B—C6B—C5B	−1.2 (2)
C2A—C1A—C6A—N1A	−173.81 (13)	C2B—C1B—C6B—N1B	−177.79 (13)
N3A—C5A—C6A—C1A	179.38 (14)	N3B—C5B—C6B—C1B	−176.31 (14)
C4A—C5A—C6A—C1A	−3.9 (2)	C4B—C5B—C6B—C1B	0.6 (2)
N3A—C5A—C6A—N1A	−4.4 (2)	N3B—C5B—C6B—N1B	0.3 (2)
C4A—C5A—C6A—N1A	172.33 (13)	C4B—C5B—C6B—N1B	177.25 (13)
C7A—N1A—C6A—C1A	−77.9 (2)	C7B—N1B—C6B—C1B	−50.4 (2)
C7A—N1A—C6A—C5A	105.78 (17)	C7B—N1B—C6B—C5B	133.02 (16)
C6A—N1A—C7A—C12A	−0.5 (2)	C6B—N1B—C7B—C12B	−7.7 (2)
C6A—N1A—C7A—C8A	−178.66 (14)	C6B—N1B—C7B—C8B	170.43 (14)
N1A—C7A—C8A—C9A	−152.68 (14)	N1B—C7B—C8B—C9B	154.69 (13)
C12A—C7A—C8A—C9A	29.1 (2)	C12B—C7B—C8B—C9B	−27.1 (2)
C7A—C8A—C9A—C10A	−50.05 (18)	C7B—C8B—C9B—C14B	169.43 (13)
C7A—C8A—C9A—C13A	−170.00 (13)	C7B—C8B—C9B—C13B	−70.59 (17)
C7A—C8A—C9A—C14A	70.27 (17)	C7B—C8B—C9B—C10B	49.73 (17)
C13A—C9A—C10A—C11A	167.20 (14)	C14B—C9B—C10B—C11B	−169.64 (13)
C8A—C9A—C10A—C11A	48.27 (18)	C13B—C9B—C10B—C11B	69.49 (17)
C14A—C9A—C10A—C11A	−71.94 (18)	C8B—C9B—C10B—C11B	−50.92 (17)
C9A—C10A—C11A—O1A	157.90 (14)	C9B—C10B—C11B—O1B	−151.53 (14)
C9A—C10A—C11A—C12A	−24.2 (2)	C9B—C10B—C11B—C12B	29.4 (2)
N1A—C7A—C12A—C11A	−179.87 (14)	N1B—C7B—C12B—C11B	−179.99 (14)
C8A—C7A—C12A—C11A	−1.8 (2)	C8B—C7B—C12B—C11B	2.0 (2)
O1A—C11A—C12A—C7A	176.94 (14)	O1B—C11B—C12B—C7B	177.82 (14)
C10A—C11A—C12A—C7A	−0.9 (2)	C10B—C11B—C12B—C7B	−3.1 (2)

### Hydrogen-bond geometry (Å, °)

**Table:**

*D*—H···*A*	*D*—H	H···*A*	*D*···*A*	*D*—H···*A*
N1A—H1NA···O1B^i^	0.89 (2)	1.99 (2)	2.8678 (17)	172 (2)
N3A—H2NA···O1A^ii^	0.90 (2)	2.09 (2)	2.9874 (19)	176 (2)
N3A—H3NA···O2B^iii^	0.91 (2)	2.40 (2)	3.258 (2)	156 (2)
N1B—H1NB···O1W^iv^	0.89 (2)	2.45 (2)	3.2651 (15)	152 (2)
N3B—H2NB···O1B^v^	0.88 (2)	2.05 (2)	2.9161 (19)	169 (2)
N3B—H3NB···O1A^vi^	0.88 (2)	2.06 (2)	2.8963 (18)	159 (2)
C8A—H8AA···O2B^iii^	0.97	2.55	3.280 (2)	132
C8B—H8BA···O1W^iv^	0.97	2.32	3.247 (2)	160

Symmetry codes: (i) *x*, −*y*+1, *z*+1/2; (ii) −*x*, −*y*+2, −*z*+2; (iii) *x*, *y*+1, *z*+1; (iv) *x*+1/2, *y*−1/2, *z*; (v) −*x*+1/2, *y*+1/2, −*z*+1/2; (vi) −*x*+1/2, *y*−1/2, −*z*+3/2.
